# Sexist advertising of women car washers in the Andean mountains

**DOI:** 10.3389/fsoc.2025.1442815

**Published:** 2025-04-08

**Authors:** Edgar Gutiérrez-Gómez, Sonia Beatriz Munaris-Parco, Ketty Marilú Moscoso-Paucarchuco, Juan Quispe-Rodríguez, Jaime Carmelo Aspur-Barrientos

**Affiliations:** ^1^Escuela de Administración de Turismo Sostenible y Hotelería, Universidad Nacional Autónoma de Huanta, Ayacucho, Peru; ^2^Área Ciencias Sociales, Institución Educativa “Nuestra Señora de Fátima”, Ayacucho, Peru; ^3^Departamento de administración, Universidad Nacional Autónoma Altoandina de Tarma, Junín, Peru; ^4^Escuela de Ingeniería de Gestión Ambiental, Universidad Nacional Autónoma de Huanta, Ayacucho, Peru

**Keywords:** machismo, misleading advertising, sexism, car wash, Andes, sierra

## Abstract

The struggle for gender equity is progressing positively; however, the Peruvian highlands, characterized by its predominantly macho characteristics, lags behind in achieving this equity. The study on sexist advertising in car wash centers in the Peruvian highlands was conducted based on participant observation and interviews with the main actors who display sexist advertising posters on public roads. The objective of this study is to analyze the sexist implications of advertising posters with images of young women in skimpy clothing, exposing their body parts in full color while sensuously bathing in water, soap, and shampoo foam. This serves as an advertising hook to attract male customers seeking car washing services. However, it is concluded that this form of illegal advertising and labor practice is deemed sexist, as it lacks prior control by local authorities and violates principles of individual privacy and ethics. Sexism in car washes in the Peruvian highlands reaffirms the macho behavior of both private and public car drivers.

## 1 Introduction

The labor situation in Peru is marked by a high level of informality at the national level, leading to jobs such as car washing being carried out informally and often reflecting a clear sexist tendency. This reality is especially worrisome, as “this problem is especially acute in rural areas, where it reaches 95.3% compared to the 70.5% recorded in urban areas” (Comexperu, 2023). This research was conducted in a vulnerable rural area of Peru. In addition to the informal work environment, there exists a sexist society which uses phrases such as “you look prettier when you are quiet,” “she who does not teach does not sell,” “you should have stayed cooking,” “you are an automatic taller.” These are some of the sexist phrases that are more prevalent against women and that many people have heard (Cabeza, 2024). In the Andean highlands of Peru, the climate is cold due to the high altitude. The research site is in the region of Ayacucho at an altitude of 2,761 m, located in the southern part of the Andes Mountains. Sexist advertising in car wash shops, featuring images of half-naked women, contradicts the image of Ayacucho women. The Andean highlands, which is the location of the research, reports alarming cases of violence against women: “the Demographic and Family Health Survey (ENDES) of 2022, the percentage of cases registered in the region exceeds 50%, representing the number of women who have been victims of some type of violence by their partner” (Quispe, 2023a).

Car wash centers are located at strategic points in the city; in Ayacucho, they are found along the highways leading to other regions. Ayacucho is connected by a road to the west with the capital, Lima, via the Via Los Libertadores highway; to the east with Huancayo; and to the south with Cusco. These areas are plagued by posters with images of half-naked women on public roads, where advertising space is limited: “the female body is used, because of its attractive force, to capture attention” (Quirós, 2020, p. 360). The full-color images of semi-nude women, which highlight their erotic features, do not have authorization from the person depicted in the poster, and they do not carry their names; according to the car wash owners, these images are downloaded from the Internet. The owners of the car wash establishments prepare the environment for male clients, despite the fact that in Peru, “women drivers represent 15% of the total number of people with driver’s licenses for vehicles with four wheels or more” (Gob.pe, 2024). Paradoxically, more women work in car wash centers, which is at odds with the sexist images often seen in advertising. The workers in these informal establishments bear no relation to the half-naked bodies that appear on the billboards. In addition, the staff includes children and Venezuelans who have migrated to Peru and are stigmatized as “a family passing by with their vehicle at the intersection of Angamos Avenue and Paseo de la República had a distressing time after they refused to let a group of Venezuelan citizens clean the windshield” (Infobae, 2022).

Informal work in Peru has no police backing or state support in its development, car wash centers can often turn into brothels, canteens, robberies, scam centers, or bars with flashy signs featuring a young woman in revealing clothing. Similar incidents happen in other parts of the world: “during the day women were forced to wear suggestive clothes to wash customers’ cars” (Times, 2016). In this research, women are not the ones washing cars in suggestive clothing. Instead, the signage in almost all facilities is suggestive. In the car wash shops, the work is done by women, men, and children, which contradicts the advertisement’s portrayal, “One of the most common practices in advertising since its beginnings has been to place women as an “object” within all advertising content; women are portrayed without personality, without their own identity, only offering their body and beauty for the satisfaction of men” (Gordillo, 2008, p. 406). In Peru and the Peruvian highlands, a macho culture prevails that discriminates against working women who do not possess a body like that of the car wash images: “women have assumed the leading role that corresponds to them in society and this role was also transferred to the automotive market, a segment that years ago seemed to be dominated by the male public” (Redacciónperu21, 2015a). These car wash centers are also frequented by women who drive their vehicles.

The research found no posters of portly males compared to the professional models in the billboards. The car wash billboards have different models that suggest sexism and eroticism. Generally, they found: “the image of a part of the female body (specifically hips and buttocks of a woman) in a disproportionate, excessive and, consequently, unjustified way, by showing the woman in the action of undressing completely” (Vidal, 2013, p. 42). In the Andes of Peru, due to its topography, the climate is frigid for most of the time. This advertising does not reflect the life of women in the countryside who managed to gain space. For example, “Gloria Ccaico Zarate is the first female Varayoc to have held the position in the Ccarhuaccocco community” (Gutiérrez-Gómez et al., 2023a, p. 4). In this research, we analyze sexist and misleading advertising that lacks prior control and supervision by the authorities. There is no law protecting women from the exploitation of their bodies in car wash shops, many of which operate in unsanitary conditions without supervision from the relevant authorities.

## 2 Methodology

This qualitative research is based on observation, participant observation, and interviews with the main actors involved in the car wash business along the entrance and exit roads of the city of Ayacucho. The information processing involved a content analysis and narrative approach based on data collected from the actors found along the three main arteries that connect the city, where informal car wash shops are located. These spaces are marked by billboards displaying images of young women, with contoured and semi-nude bodies of different sizes.

The researchers acted as pedestrians, making several rounds at the three car wash locations. The investigation found that the traffic was chaotic due to the advertising on the roadway, the cars, the car washes, and the splashing of water when washing the cars. On other occasions, we have rented cars with different characteristics and have been customers, where the service is different and access is easier, such as the price of the wash. Generally, there are three prices for the characteristics of the vehicles; however, a special price applies to tourists or outsiders who are from the capital and the price is higher. It was necessary to go to the car wash shops on several occasions to participate as observers; information about their operating license, the use of potable water, electricity, Venezuelan workers, and images of half-naked women is hermetic. The Submanagement of Tax Administration, Authorization of Commercial Establishments of Operating License of the Municipality of Huamanga reports that there is no regulation for licensing a vehicle washing center, it is prohibited to use drinking water.

In the intermediaries of the car wash centers, the trade of lubricants is intermingled in the same way with images of women in revealing clothing; in that section, there are also tire sales centers exposed on public roads that do not allow pedestrians to circulate freely. As researchers, we used the forms of participation in observation as pedestrian, participant observation, and interviews with customers, workers, and owners. The language they use in their communication is coprolalia, they always have a rag in their hand once they have finished their work. In the data collection stage, no supervisor or worker from the municipality of their jurisdiction, which belongs to three districts, was present. With the information collected, we proceeded to the processing, with recurring themes of sexism, images of women in revealing clothing, suggestive positions of photos taken from the Internet, exposed in the form of anonymity, perfect bodies of young women sprayed with water and shampoo foam with soap, accompanied in bottles of beer.

## 3 Results

### 3.1 Image of women in advertisements at car wash facilities

**Figure 1:**
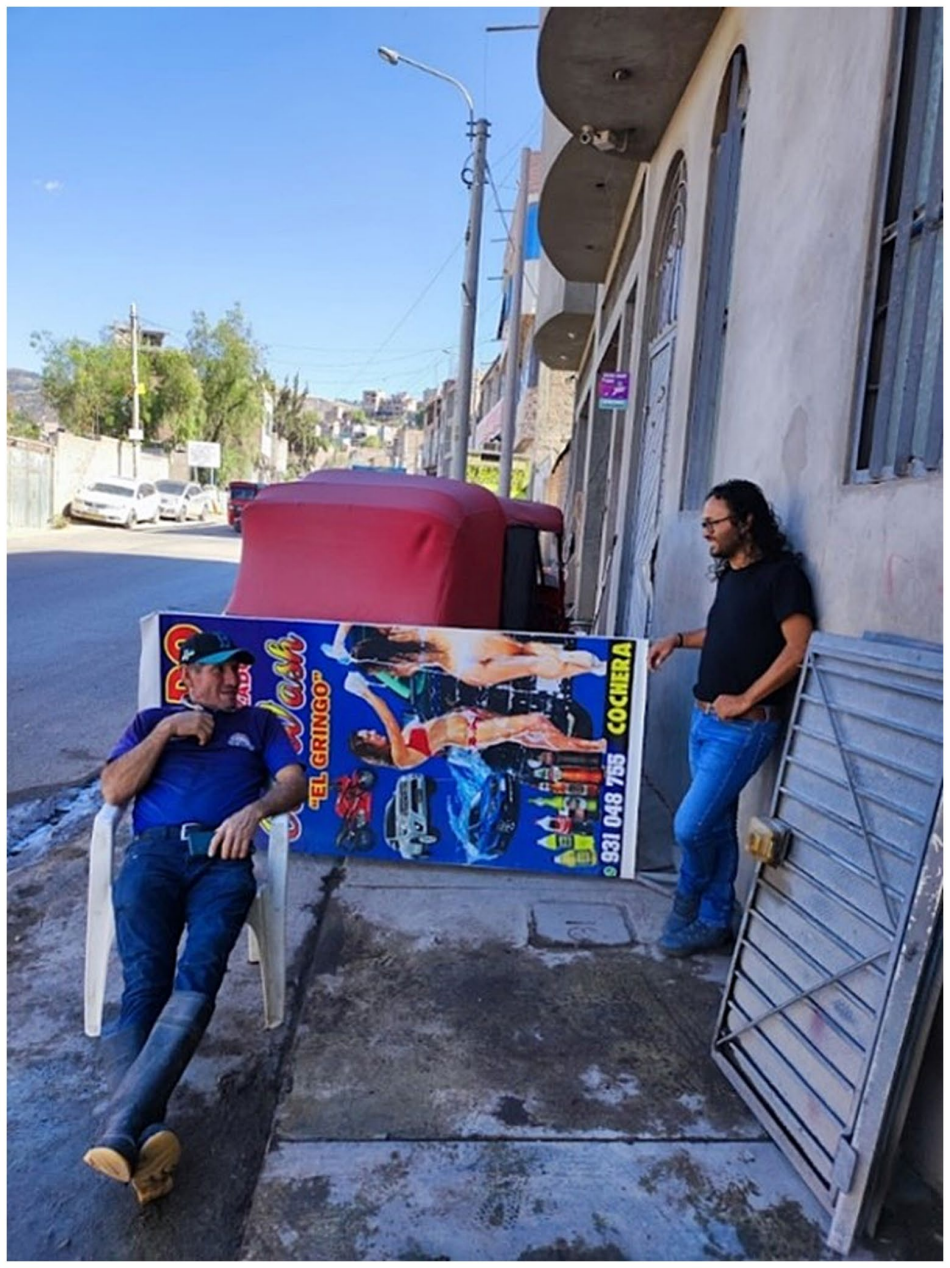
The two images of women in tiny clothes are displayed on the public road at the exit of the highway to the east, on the road to Huancayo. The middle photo is the interior of the car wash, where there is a space to drink liquor and play music to the taste of the client on duty. Source: Photo by the authors May 2024.

The indiscriminate use of images featuring scantily clad young women in sexist advertising at majority of the car wash centers goes unchecked. At the exits of the Ayacucho interprovincial highway, car washing work is carried out informally on public roads. The outstanding example of a few women in relation to the world of cars, such as “‘the first and only woman in Peru to drive an Enatru bus,’ is left behind. Those that until the early 1990s, carried 300 passengers and were much larger than the Metropolitano cars” (Redacciónperu21, 2015b). It is a social phenomenon that women are entering the automotive world, a sector dominated by men. The majority of the centers, such as car wash shops, have on their doors or the street the “car wash” sign with the image of a woman dressed in short and suggestive clothes (Figure 1).

The quality and preservation of sexist advertising posters are displayed on public roads for full view for everyone, testing the patience of passersby. The exhibition lacks control; there is no supervision, no fines, and no law prohibiting this advertising. The photos lack a description of who the woman is, if she authorized them, or if they are simply the result of image theft: “that of placing the woman as an ′object′ within the entire advertising content; the woman is without personality, without her own identity, she only puts her body and beauty at the service of male satisfaction” (Gordillo, 2008, p. 405). The majority of the customers are men who are drawn in by advertisements featuring women due to their feminine beauty. Many car wash services are located on public roads, where pedestrians walk, occupying the entire berm in high vehicular traffic and endangering pedestrians’ lives. The exits to the east, west, and south of Ayacucho are crowded with car wash centers, making pedestrian traffic almost impossible because in each place there are “jaladores” (looking for customers) with thuggish behavior. It is forbidden to move their signs when passing: “sexual women are represented within the prototype of current beauty, and although they do not appear naked, their very tight clothes let intuit the female body and on several occasions they appear wet, a detail that further emphasizes the eroticism of the female figure” (Muñoz-Muñoz and Martínez-Oña, 2019, p. 1134). The so-called jaladores intimidate pedestrians; there is no control or order to get a customer.

In the historic center, there are commercial “car wash” stores that offer guaranteed services, but they still use images of half-naked women in their advertisments. These stores cannot compete with the informal car wash centers located on public streets, which charge between US$3, US$5, and US$7, depending on the exchange rate. These laundromats do not pay taxes, have clandestine water connections, and steal electricity from high-tension wires. Under these conditions, the payment for each car wash service is lower for women than for men and varies based on the number of cars each worker washes during the day. The customers arriving at the car wash service are approached by several young men offering a better service and showing their sign: “serving the female body as a sexual lure, but directly or indirectly also inculcate a conception of sex that on countless occasions presents women as merchandise” (Carretero, 2014, p. 142). The competition also involves which gas station has the best ambiance, offering complementary elements such as perfume, a poster of a woman with a sexist body, and a place to drink liquor while washing the car.

The municipal authorities in charge of their jurisdiction do not control or supervise the illegal display of images of anonymous women with sexist semi-nude bodies. Car wash services are carried out with potable water despite the water scarcity in the Ayacucho region. The billboards displayed on the public streets where the car washes are conglomerated look like advertisements for a “Pink” sexual services area. There is no adequate control and any passerby can see an exhibition: “degrading for the person, male or female, by conferring the body the status of simple sexual merchandise, has led to prohibit its advertising in countries such as Germany, France, Portugal, etc.” (Quirós, 2020, p. 359). Other countries prohibit the display of sexist images of women to attract male customers who own cars and require washing and cleaning services. Every morning when they open their car wash store, workers take the posters out onto the street and put them away again in the afternoon. These stores are run by businessmen who have more than one store at the three highway exits in the city of Ayacucho. The problem of water scarcity in the Andean region continues to be a latent concern, along with informality and the lack of control by the authorities in these sexist work zones. In the region, there are reports of “Ayacucho has a total of 28 districts in 7 provinces placed in emergency” (Quispe, 2023b).

**Figure 2:**
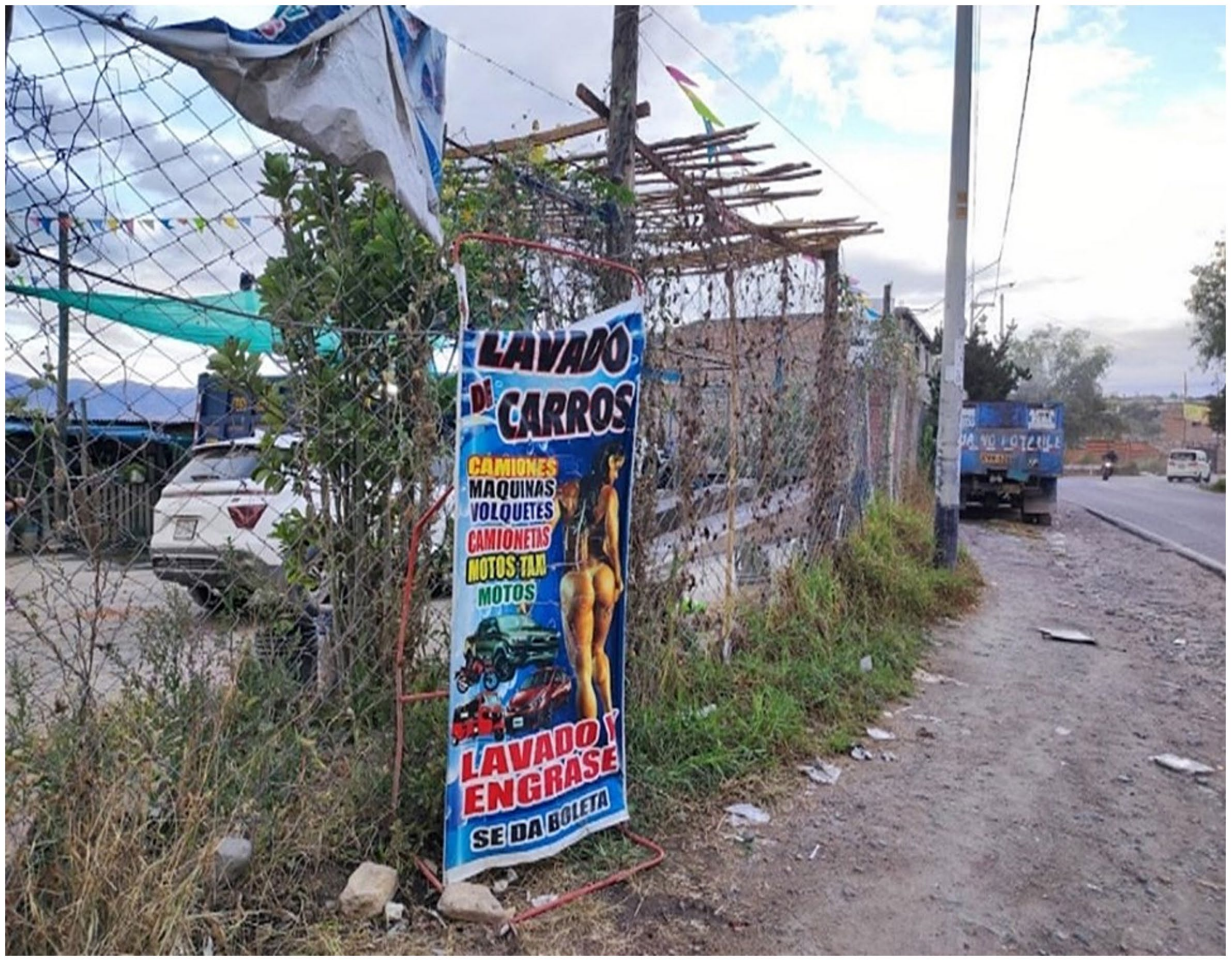
Posters on the public road, the interior of the space is improvised. The posters show a half-naked woman. The owner agrees to a photograph with one of the researchers. Source: Photo taken by the authors, May 2024.

People working in carwash establishments are interested in attracting customers and care little about passersby on the pedestrian berm. Their attention is focused on the vehicles on the highway that are potential customers. The streets are filled with billboards featuring women in revealing clothing that catch the attention of men and captivate them: “the use of the perfect female body; the image of women as an aesthetic and/or sexual value (women do not perform any function, they are simply an ornament or advertising claim) and the image of the ‵superwoman′” (Victoria et al., 2006, p. 78). The workers, who do not appear to be owners of the car wash business, behave aggressively with people who want to learn how the car wash system works, with images of half-naked women. Still, other people work inside, which is not consistent with the sexist advertising. The interior of the few with space for more than one minor vehicle to enter, because almost all of them provide service on public roads, is completely unhealthy. There are no sanitary guarantees, because municipal authorities do not supervise them as they are illegal businesses: “advertising is a relevant activity in competitive contexts. It fulfills important functions and requires to be developed under ethical principles of truthfulness, integrity and social responsibility” (Zapata and Tejeda, 2016, p. 232). The exterior of the premises, where the image of a young woman dressed in tiny clothes is displayed, does not contrast with the interior of the premises (Figure 2).

### 3.2 The physical structure of the workers in the facilities does not match the figure on the billboard

Interviews with passersby, clients, and neighbors of car wash services, mostly Venezuelans who work illegally and earn according to the number of vehicles washed daily, show that the reality of the work does not match the advertising images. An investigation shows that “they chose Peru and the city of Ayacucho as places of arrival driven by the positive comments of stability, ease of getting work and earning money; generating social imaginaries of work, money, self-improvement, quality of life and well-being” (Martínez, 2019, p. 177). The work they were able to access consisted of washing cars in informal places that were not supervised by local or national authorities. The work is done on public roads with limited space for a small vehicle, and posters featuring attractive women are also displayed along these roads. Venezuelan workers, peasants, children, and other age groups do not match the advertised image. Work cases such as car washing for mothers: “it is about a mother carrying her baby in a lliclla (Andean blanket) and this does not stop her from working in a car and motorcycle washing area in that jurisdiction” (Ticona, 2022). There is no place where the car wash service is advertised for women on the sign.

In the majority of the cases, there is no relation between male customers driving public and private service vehicles and their visits to car wash centers due to flashy posters featuring women in revealing clothing. With the passage of time and the inclement weather and heat, the posters are in constant deterioration and renovation by the owners, justifying: “the use of the female body as a mere object, which, seems to allude to the use of said image as a purely decorative element” (Quirós, 2020, p. 261). This decorative element violates the right to privacy and gender equity the Peruvian State promotes. The images of women with bodies stereotyped by the current canons of beauty violate and discriminate against Andean women in geography of: “low temperatures, especially in the high Andean areas of Ayacucho, Arequipa, Moquegua, Puno and Tacna, in the southern highlands of Peru, where values of up to 15 degrees below zero are expected” (Castro, 2014).

In interviews with some commercial car wash owners, they stated that they hired young women with slender bodies to attract customers. The images on the billboards are taken from the Internet; it is not known who the woman in the photo is; they made some arrangements with beer and soft drink bottles to advertise their premises. The advertising is misleading as a hook for the male customer who drives a car and needs morbidity: “the woman is used as a weapon of seduction to consumption. The beauty of the woman with a body is the center of attention in advertisements” (Del-Moral-Pérez, 2000, p. 214). This concept is corroborated by the hundreds of images found on the street during the research; some deteriorated over time and weathering, and others were freshly made. In the interviews with the owners, many agree that having these images and women who come, especially when the store opens, is a perfect hook to attract customers. Once loyal customers come for the good service provided the posters with half-naked women are already decorative, as the interviewee states.

Regarding the control of the operating licenses of the premises, they state that they are in the process of acquiring their license from the Provincial Municipality of Huamanga. When the supervisors come, which they rarely do, they are informed that the license is still in progress. In some instances, a few bribes are offered, and the supervisors leave without proper follow-up. In this sense: “the institutionalization of the problems continues to be nefarious, the institution persists erroneous from its base and public policies, therefore, have not stopped walking but towards a control of the bodies of us women” (Ibáñez, 2014, p. 223). The posters distributed in different posts that lack a firm base constantly fall down due to the winds of the Andean highlands. The sidewalks which are frequented by passersby, are invaded by these posters advertising car wash services, in the sector of the exit to Huancayo in the east of Ayacucho, where more Venezuelans work, who are dedicated to crime, according to those who have car wash shops in the west of Ayacucho, at the exit to Vía Los Libertadores. They say that winning customers requires a good image of seductive women: “advertisements that represent women in a vexatious or discriminatory way, either by using particularly and directly their body or parts of it as a mere object disconnected from the product to be promoted, or their image associated with stereotypical behaviors” (Carretero, 2014, p. 133).

Water scarcity in the highlands of Peru is a constant problem; car washes operate with potable water, some even using clandestine connections, which adds to labor informality and the prevalence of sexist advertising. The company SEDA (Servicio de Agua Potable y Alcantarillado de Ayacucho S.A.) has a slogan: “water is the sustenance of life, it is a limited resource, with our habits and activities we are contaminating it. We should all help to conserve it and use it properly with simple actions” (SEDA, 2024). The slogan is decorative, and, in practice, they do not sanction illegal car wash workers. Those who work at the Vía Los Libertados exit, west of Ayacucho, do not want Venezuelans, because they engage in criminal behavior and steal from their clients. The advertising is totally misleading, in none of the premises where women wash cars with revealing clothing: “misleading is in the receiver, in the distance with other antecedents, or in the discrepancies with the service finally provided. For the same reason, it is difficult to sustain *a priori* whether or not the advertising held in view constitutes misleading advertising” (Zapata and Tejeda, 2016, p. 228). There is no correspondence between workers and women in Ayacucho; in fact, there are more men than women among workers.

As the vehicle washing work is carried out on the public road, occupying the sidewalks for pedestrian traffic, the place is not welcoming. It has reduced spaces for customers who can consume liquor and soft drinks purchased from the owner of the premises, while washing their vehicles. The common sense of the neighbors and passersby is normalized to: “represent the female body as an object, as an added value to the attributes of a certain product; in short, as its packaging” (Victoria et al., 2006, p. 82). When they finish the work of one client, they are waiting for another, making gestures of pulling with a rag in their hand toward their workplace, which is the public road. This research does not analyze the type of work or the way they wash the car, but the sexist advertising of their posters in the middle of the street: “the image of women in advertisements has moved from their traditional role of mother and wife to another based on the sensuality of stylized, perfect and unreal bodies” (Montes-Vozmediano and Torregrosa-Carmona, 2018, p. 438). There is no relationship between the service, the advertising, and the workers—women, children, and men—who are paid a daily wage for each car wash.

### 3.3 The informality of sexist advertising and the work of car washers

Informal work in Peru and the Andean highlands is evident and are not controlled by the authorities: “According to the International Labor Organization (ILO) 127 million Latin Americans, 47% of the labor market, belong to this category” (Justo, 2014). They do not pay taxes on their income and workers do not have life insurance. When they first open their premises, they look for a bold woman to attract customers. Still, as time goes by, their work becomes unsustainable, because their work is unsustainable, as our interviewees stated. The informality of the work in the car wash centers, with flashy and sexist signs, generates insecurity, such as robberies: “the alleged group used the car wash facilities as a hiding place for those cars that were stolen from their legitimate owners” (LR, 2020b). This information is corroborated by workers in the western and southern zones of Ayacucho, who accuse those in the east, who are Venezuelan, of being thugs and criminals. Driving on streets without sidewalks creates insecurity for pedestrians, especially due to the invasion of sexist posters and cars being washed on high-traffic roads: “presenting the image of a woman as an object to capture their attention, with respect to certain types of products aimed at the male public” (Vidal, 2013, p. 44). Driving cars is related to the male sector and is the justification for sexist advertising posters.

**Figure 3:**
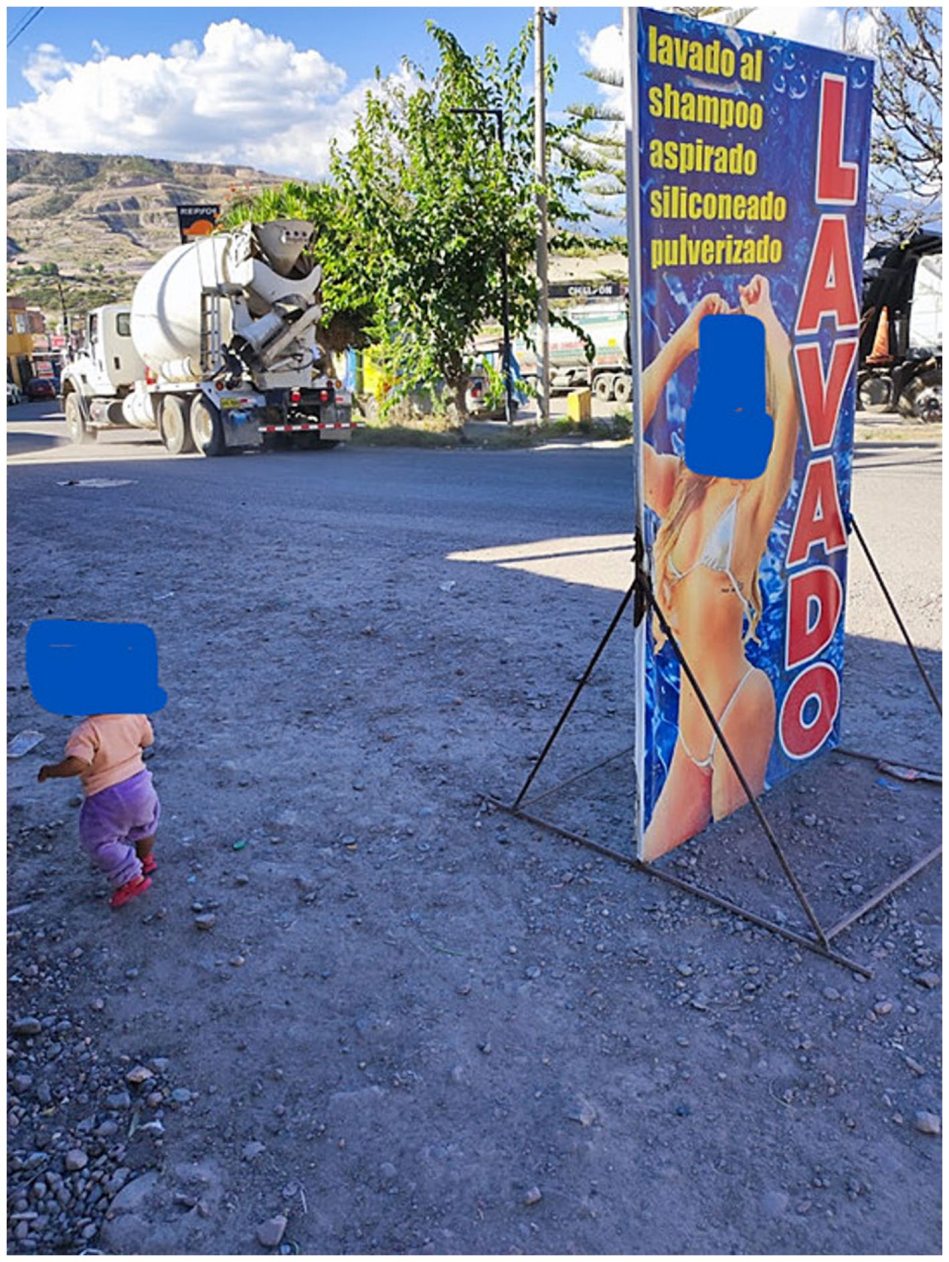
Some photos of posters on public roads and their service beside, blocked pedestrian walkway. Source: authors’ photo May 2024.

The way to approach the workers is complicated, you have to pose as one of the clients quoting the cost of the car wash. Taking unauthorized photographs is prohibited; they assume that they are supervisors from the municipality or emissaries from National Superintendence of Customs and Tax Administration (SUNAT), which can impose fines or close their premises. The work is 7 days a week; on Fridays and weekends, there is alcohol consumption among the neighbors who work informally, and cases such as the following occur: “a young man dedicated to washing cars met an unexpected death, after a recycler stabbed him in the heart and left him abandoned in the middle of the road” (Aquino, 2023). Both men and women use coprolalia in their communication. Passersby on the sidewalks invaded by images of half-naked women drenched in water and soap are constantly spurred on by the force of the water and soap suds (Figure 3).

As it is an informal work without police control or municipal authorities, these car wash services are linked to crime: “the Bravos of the Car Wash and they have taken away at least 20 vehicles in the same way. His personality is overwhelming. He easily deceived people. He gave the appearance of being a wealthy person. He even gave tips” (Redacciónperu21, 2019). Leaving the vehicle with the key inside is not a safe practice, since thefts are frequent. It is necessary to be present while the car is being cleaned. Some authorities at the national level have tried to ban it:

“The controversial decision to prohibit the activities of windshield wipers or car washers in the historic center of Lima, as announced by Mayor Rafael López Aliaga, was extended to districts such as Surco and Magdalena. This, after the murder of a driver for refusing to allow a man to offer his window washing services on Grau Avenue” (Moya, 2023).

This type of news is constant at the national level in car wash services due to their informal nature. There is not enough space in the Peruvian highlands to offer a professional car wash service. The practice of paying workers based on the number of cars washed results in longer working hours and can lead to a loss of clients: “a neighbor denounced that an elderly woman (apparently forced), a domestic worker, washed a car outside the house where she works in the district of San Isidro during the state of emergency due to the coronavirus” (LR, 2020a). This type of journalistic report is constant; a percentage of each car wash is for the worker, and there is no fixed monthly salary; it is a daily payment for the number of vehicles washed. Sexism in these premises is vast and largely unchecked: “the difference between feminine and masculine genders added to a discriminatory factor due to such difference, also including homosexuals, transsexuals and hermaphrodites is considered sexism” (Moreira, 2012, p. 5).

The need for work forces them to accept the conditions proposed by the owners of these car wash shops: “its role in society and contemporary housing in Latin America, there is now greater gender equality and an increase in women’s participation in the labor force” (Echeverria-Bucheli and Carlos-Alberto, 2023, p. 3). The municipal authorities who visit these car wash conglomerates only suggest that they build drains and not let the water run into the road. Car washes invade several blocks, each one with its own sexist sign that generates danger: “delinquents entered a car wash center and stole more than S/. 70,000 in machinery. The incident occurred in the early hours of Thursday, October 8, on Próceres de Huandoy Avenue, in the district of Los Olivos” (LR, 2020c). Some car wash shops have sophisticated machinery installed on public roads, while others have rustic machinery that depends on the acquisition and investment capacity of their business. All workers work without personal safety protection, use detergents without protection, and use a piece of cloth with a stamp indicating that it is a car wash service.

### 3.4 Male clients in a stereotypical advertisement in the Andean highlands

The physical characteristics of the Andean inhabitant differ abysmally from the sexist advertisements that car washes use in their advertising. It is a deep-rooted tradition in the region of Ayacucho, with its capital Huamanga, and is known as the huamanguina woman with her typical costume: “of huamanguino, men wear a blue jacket, white shirt and hats; while the ladies wear a white hat with blue blouse, white blouse, petticoat, skirt, skirt, center, and other clothing” (Quiroz, 2014). Among the inhabitants of the Peruvian highlands, the feeling that using typical costumes is a regional identity is deeply rooted. The marked sexism of advertising blurs the female situation: “Peru women are about 16,640,000 and would represent 50.4% of the total population. In addition, their participation in economic activity has registered advances in recent years” (Ticona, 2022). Incorporating women into the labor market in Peru is a progressive advance that is not reduced to exposing their bodies as objects of attraction or business.

The interviewees, who gave access to a conversation, have allowed us to determine that customers are mainly from the male sector. The purpose is to feed the sexist morbidness of the customers, thus: “presents the woman in need of keeping the body beautiful and young, make it desirable, because it is the key to success in love, professional or personal; without that body 10, the woman is doomed to failure” (Quirós, 2020, p. 374). The female stereotype of the perfect body, comparable to that of television models and advertising posters, discriminates against Andean women of conservative traditions and customs. The fee for the car wash service usually varies in four prices, according to some interviewees: US$3, 5, and 7, and an extraordinary amount for new clients, who are generally tourists. This situation compares with that in other countries, such as: “in Australia, where car washing is done daily. In a video entitled ‘A day washing cars in Australia’, the young man documented his day and revealed the amount of money he earns at the end of the day” (Salas, 2023). Car washing is profitable anywhere in the world, but even more so in the Peruvian highlands due to the informality of the business and the lack of adequate spaces to rent, where the authorities show a complacent attitude toward sexism.

Gender equity and the struggle for female vindication are fundamental: “women today need to think and be rewarded not for being physical, not for being something material and not for being an object, but for the opposite” (Gordillo, 2008, p. 408). If there is no adequate control by the authorities on duty and the Ombudsman’s Office in Peru, especially in the Andes, where machismo prevails, efforts toward gender equity will be in vain. It is not a mere whim to.

“use women and their bodies reduced exclusively to a mere sexual object, passive and at the service of the sexuality and desires of men; display images of the female body or parts of it, as a resource to capture attention or as an ornament or claim, unrelated to the content of the advertisement” (Carretero, 2014, p. 139).

The use of sexist advertising in posters displayed on the road with the sole purpose of selling their product at the margin of women’s work is counterproductive.

Worldwide, sexist advertising is encouraged: “women’s bodies continue to be used as advertising claims and the phenomenon of globalization favors the propagation of these messages” (Montes-Vozmediano and Torregrosa-Carmona, 2018, p. 438). In the Peruvian highland Andes, this phenomenon of sexist advertising has been introduced, which feeds a machista tradition, as noted: “It is not known exactly when the description of a young Andean woman with a good body, described by many men as a Wira pasña, was assigned as a name to the millenary native potato” (Gutiérrez-Gómez et al., 2023b, p. 3). The Peruvian Andes have their own tradition with the ancestral Quechua language, a purely oral transmission from one generation to another. The analysis of the macho sectors is complemented: “The factor of sexism is very present due to its association with stereotypes, which are often depreciative, which ends up condemning many advertising campaigns that use them to attract attention or seek a more obvious illustration for better understanding” (Moreira, 2012, p. 6). In this sense, social and normative control of sexist advertising aimed at male customers is fundamental.

Attempts to control informality and sexist advertising, with male customers in mind, are echoed in their complaints. Some authorities try to control as is the case: “Mayor of Surco, Carlos Bruce, announced that the work of car washers in shopping malls in the district will be banned along with windshield wipers. These two activities have become not only illegal, but also dangerous” (Alomía, 2023). It is a latent danger for passersby and customers looking for a formal car wash service. It is intended to be controlled in the capital of Peru, but in the Peruvian highlands where the chaos of informality and machismo reigns: “the images of women have been mimicked with the concept of beauty and sexuality, they have become the object/subject of desire, away from qualities such as intellect” (Muñoz-Muñoz and Martínez-Oña, 2019, p. 1144). It is essential to eradicate this sexist advertising with male consumers in mind; however, in participant observation, not all male customers are interested in consuming sexist images of unknown women wearing makeup with their wet bodies adorned with soap and water on posters.

## 4 Conclusion

Illegal work at the national level in Peru is latent, especially car washing on public roads designated for pedestrian traffic. The different raids carried out by the communal authorities on the informal work of car washers do not address the issue of sexist posters: “the National Police, the Municipality of La Molina, through the Sub-management of Administrative Control, closed more than 20 premises dedicated to car washing and sale of hardware items, as well as brickworks and mechanic workshops” (EC, 2020). They are in charge of closing these commercial establishments, but within a day, they are reinstalled. In the Peruvian highlands, there is a lack of regulation; it has become a labor necessity for customers, often accompanied by images of half-naked young women. These images “violate the dignity and equality of women: presenting them as mere objects, representing them as submissive or subordinate to men, using their bodies to attract attention and attributing discriminatory stereotypes to them” (Quirós, 2020, p. 356). In an area where women from Huamanga wear different types of clothing, it is a form of underhand discrimination.

Women who work at the car wash centers, and who do not match the promoted images, limit themselves to stating that these semi-nude images, with contoured bodies and revealing clothing, are more of a hook that the owners use to attract male customers. There should be more rigorous control of sexist advertising, as it happens in Spain: “City authorities, through the General Directorate of Women, have put the case in the hands of the State Attorney General’s Office. The fact would configure before the law as “illicit advertising,” this according to article 3 of the General Law of Advertising that governs in the European country” (MN, 2019). The streets of the three intersections that have access and exit of the city of Ayacucho, the capital of the region, are invaded by sexist billboards that damage the honor of women who are unaware of their image that serves as advertising on public roads, this advertising has no direct relation to the service that is located inside the local car wash shop.

In a predominantly male society, the control of sexist advertising should be a priority and not the responsibility of the national media in the entertainment section: “feminism and machismo in entertainment and show business news. It is necessary to analyze and expose the underhanded information of the national problem of machismo and feminism in the media in the entertainment section” (Gutiérrez and Munaris, 2023, p. 2). These media feed the sexist and machista concept through misleading advertising. The woman who appears in the sexist advertising of car wash billboards is portrayed in a way that diminishes her as frivolous: “seducing the woman to get what she wants and/or awaken the desire of the man. Sometimes this model is combined with stereotypes linked to social roles such as the use of a perfect body for sexual seduction” (Victoria et al., 2006, p. 87). It is essential to enact legislation on this sexist advertising that indiscriminately uses the bodies of anonymous women in posters displayed along public roads: “In this sense, an advertising culture focused on sexism can extrapolate its moral and ethical limits in disagreement with the precepts of the law” (Moreira, 2012, p. 9). The issue of citizen coexistence on public roads is out of control. In an attempt to attract male customers, advertisements featuring female images showing their erogenous parts are being displayed.

The challenges of the investigation include controlling areas filled with car wash centers and billboards of all colors, sizes, types, and materials with images of young women in tiny clothes. The owners foster a sense of camaraderie to protect themselves from strangers who ask how to perform their car wash jobs. They often communicate with each other because their workplaces are in a drive from one place to another, in addition to commercial premises selling car lubricants and tire dealers at the same intersection. The exits mentioned in the research are traffic flows on the outskirts of the historic center. Workers receive a percentage for each vehicle washed. The informal premises are owned by small entrepreneurs who rent space and manage several establishments. The management of Servicio de Agua Potable y Alcantarillado de Ayacucho S.A. (SEDA) has complacent opinions about the informal car wash workers, claiming that they were trained to reuse water and not waste it. However, this perspective is utopian. The investigation of the indiscriminate use of potable water, the participation of Venezuelan clients and workers, and the intervention of municipal and national authorities in labor informality and the cleanliness of the city are ongoing concerns.

## Data Availability

The original contributions presented in the study are included in the article/Supplementary material, further inquiries can be directed to the corresponding author.
