# Supplementary material for: Sexist advertising of women car washers in the Andean mountains
Source: Front Sociol. 2025 Apr 8;10:1442815. doi: 10.3389/fsoc.2025.1442815 (PMC12011720; doi:10.3389/fsoc.2025.1442815)
Supplement: Supplementary file 1 [file Data_Sheet_1.pdf]

frontiers

Supplementary Material

Some web pages about Ayacucho women, their cultural tradition and identity of the people.

Web page about women in Ayacucho: https://acortar.link/5HLLeX

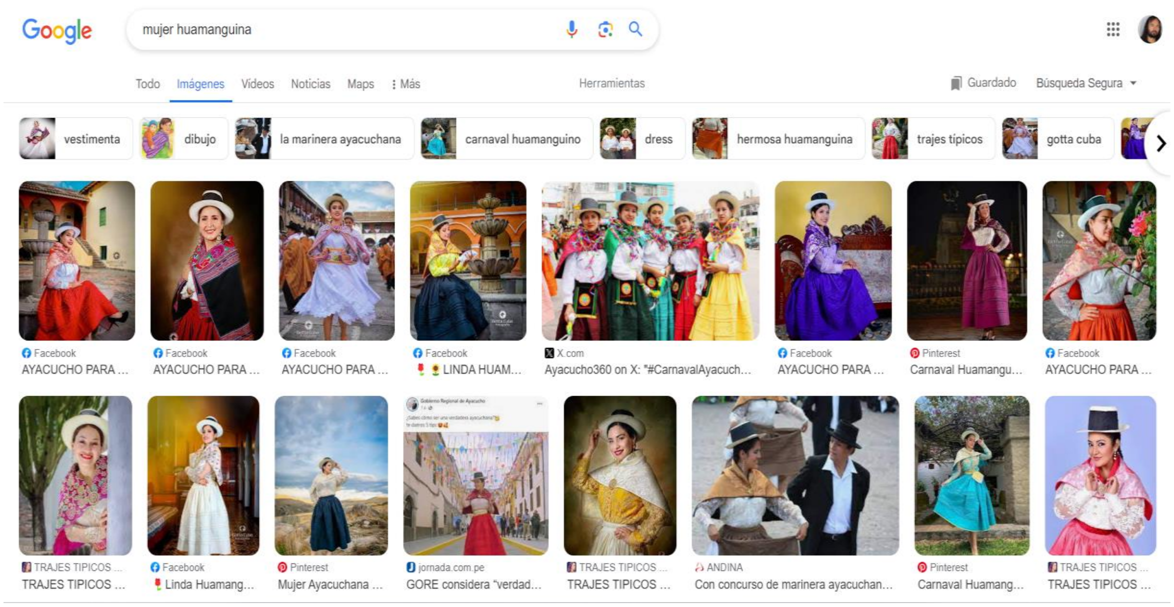

Web page about rural Ayacuchan women: https://acortar.link/l0Ossp

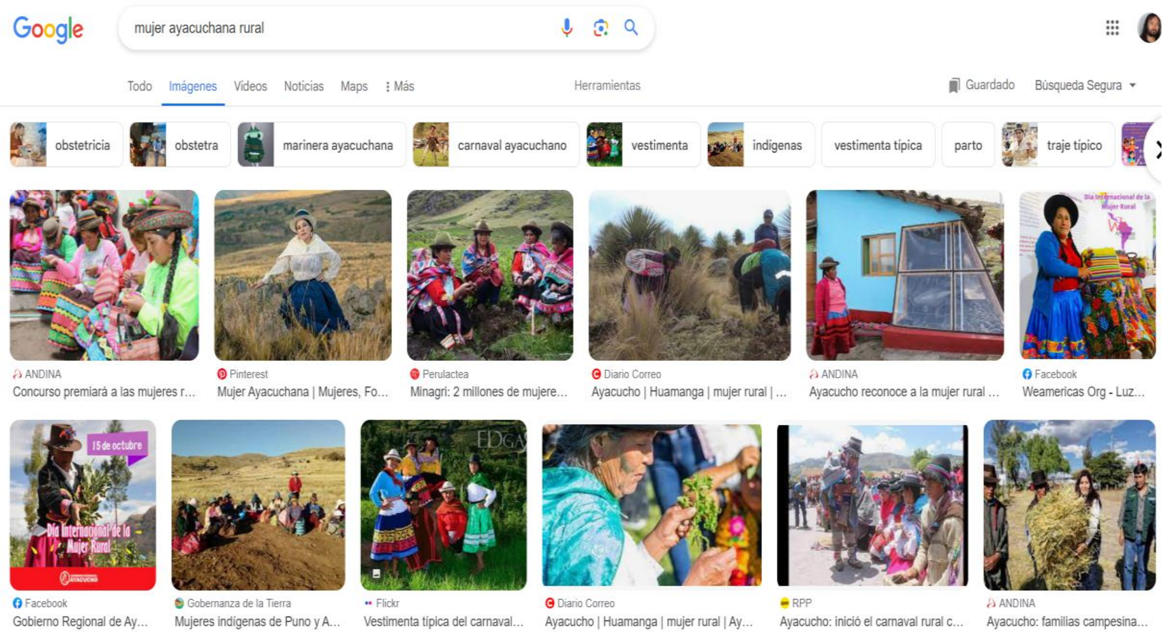

Images on the web of women washing cars Ayacucho: https://acortar.link/keRQAh

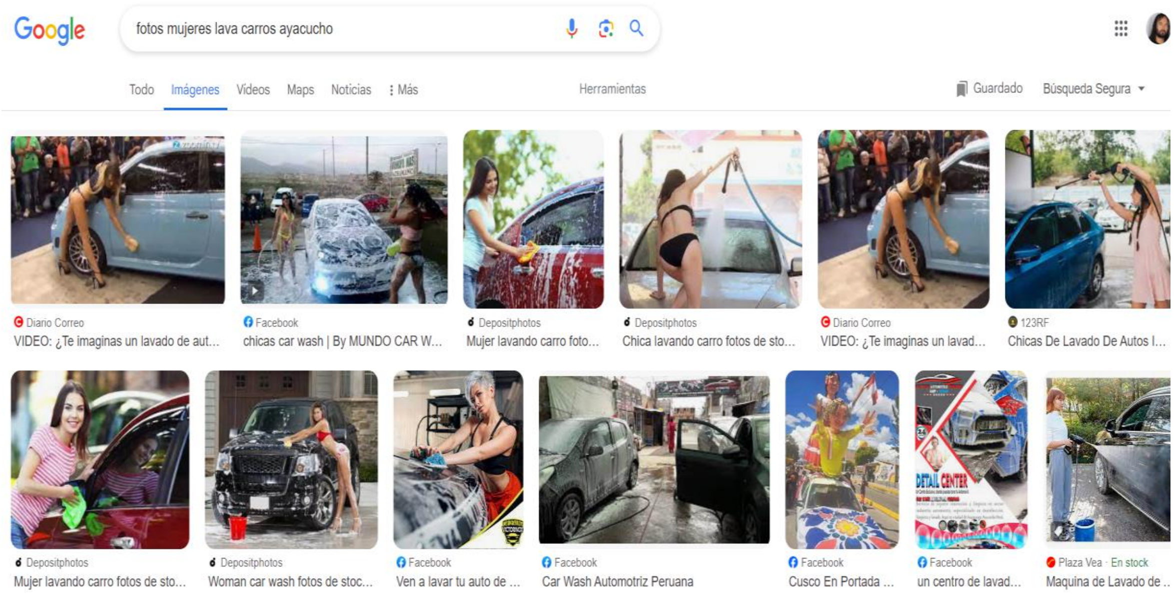

Reporte trabajo mujer: https://acortar.link/7sYAU0

¿Cuántas peruanas cuentan con brevete para manejar desde taxis hasta tráileres?

El MTC informa que en el país hay 521,439 mujeres con licencias de clase A.

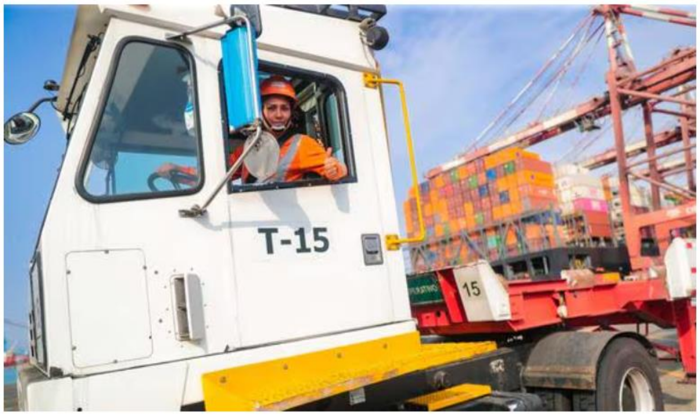

Un total de 1,479 tienen la licencia que las habilita para estar al volante de un ómnibus urbano, interurbano, panorámico y articulado, así como de remolques, grúas, volquetes y tráileres.

ÚLTIMAS NOTICIAS

Niño era torturado con cables, alicates y artefactos calientes, según reporte de...

Capturan a sujeto que golpeó hasta la muerte a carnerito 'Lilo' en Huancayo | VIDEOS

Ley de Medicamentos Genéricos: Boticas y farmacias independientes...

Attached are some photographs of hundreds of sexist advertisements on public roads in the city of Ayacucho:

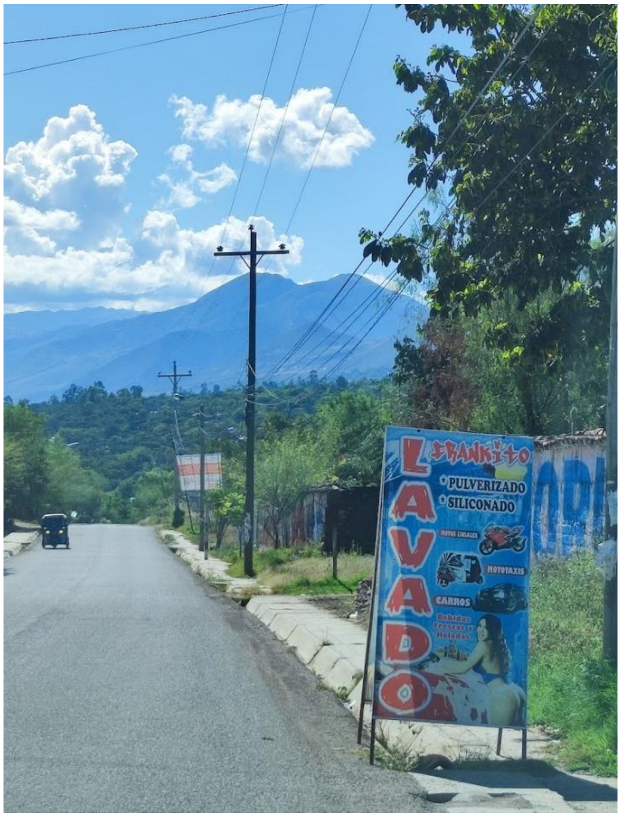

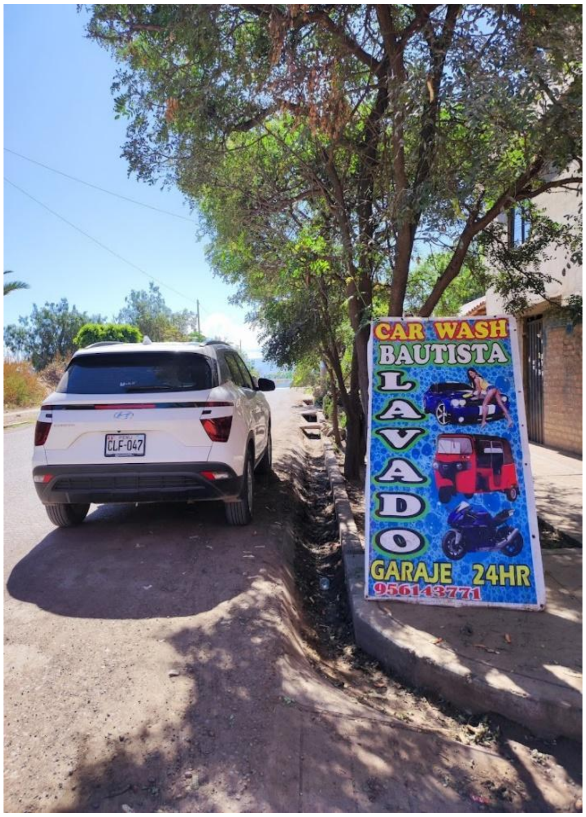

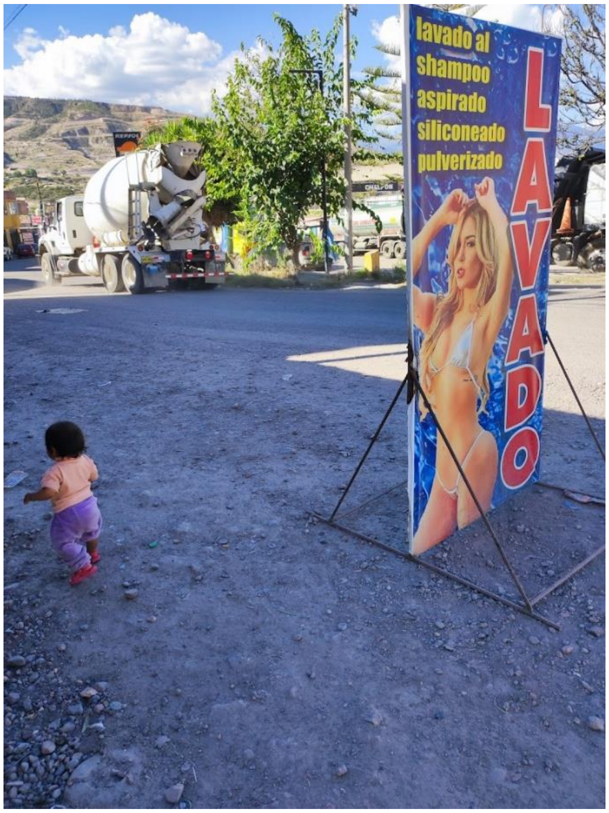

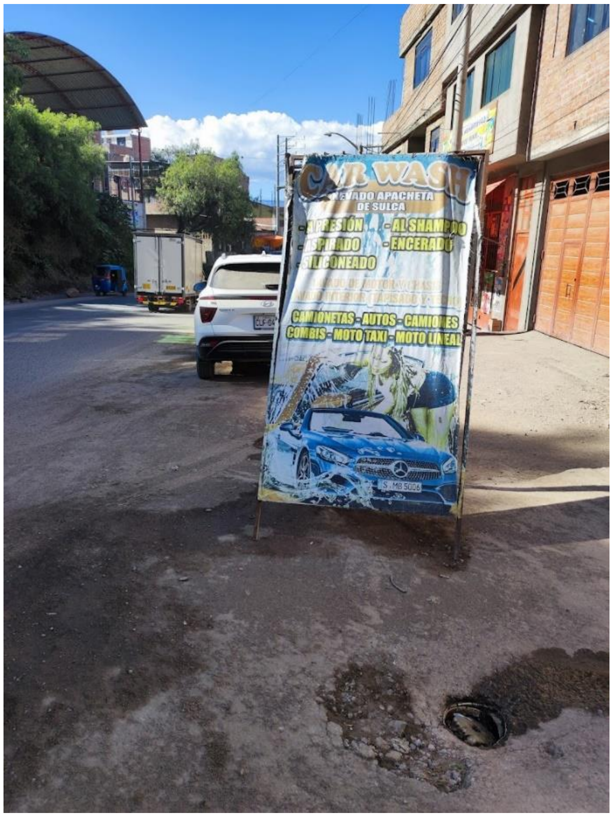

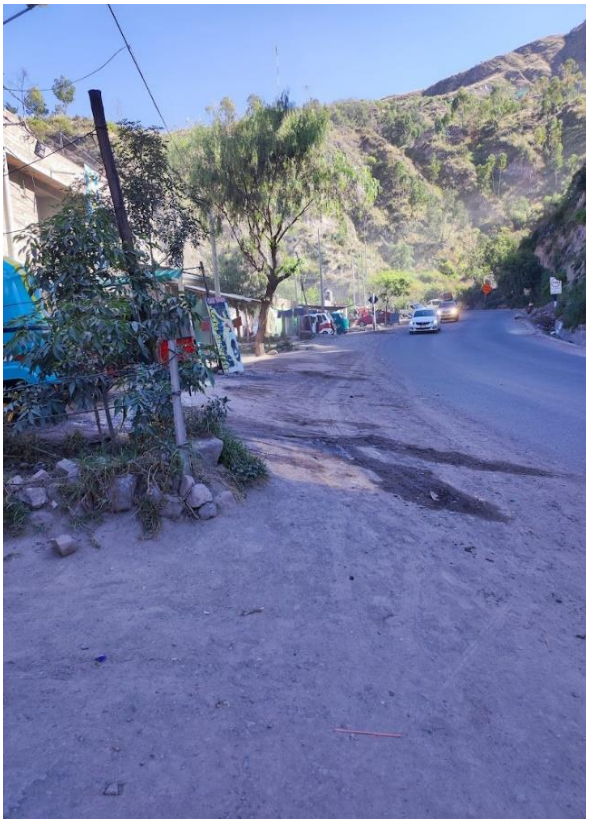

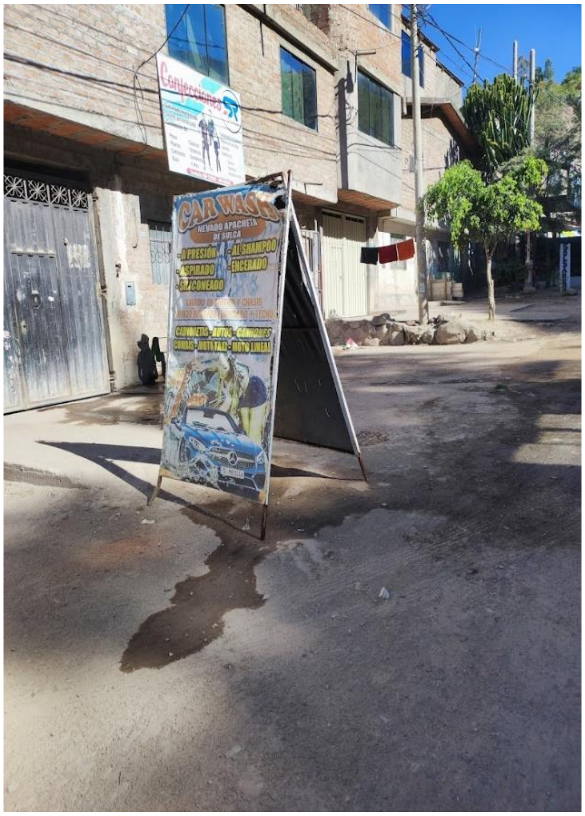
CAR WASH

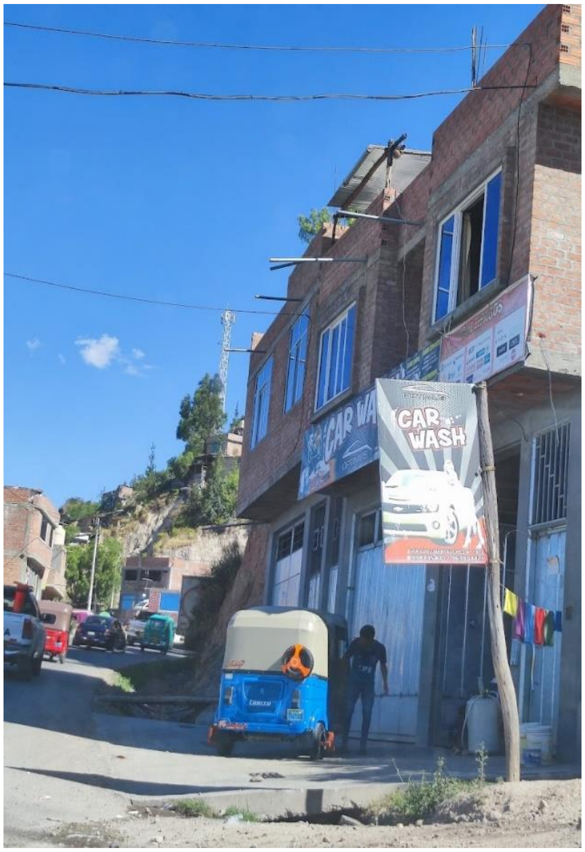
CAR WASH

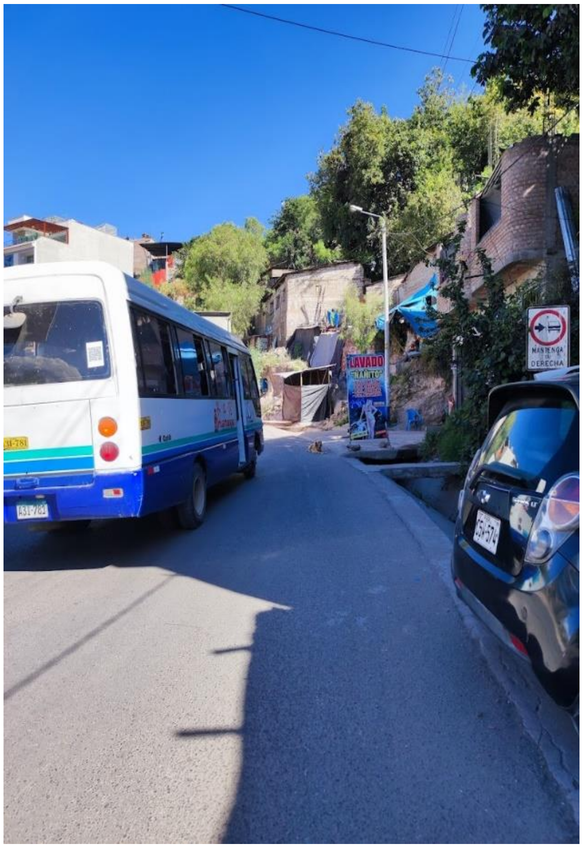
LAVADO

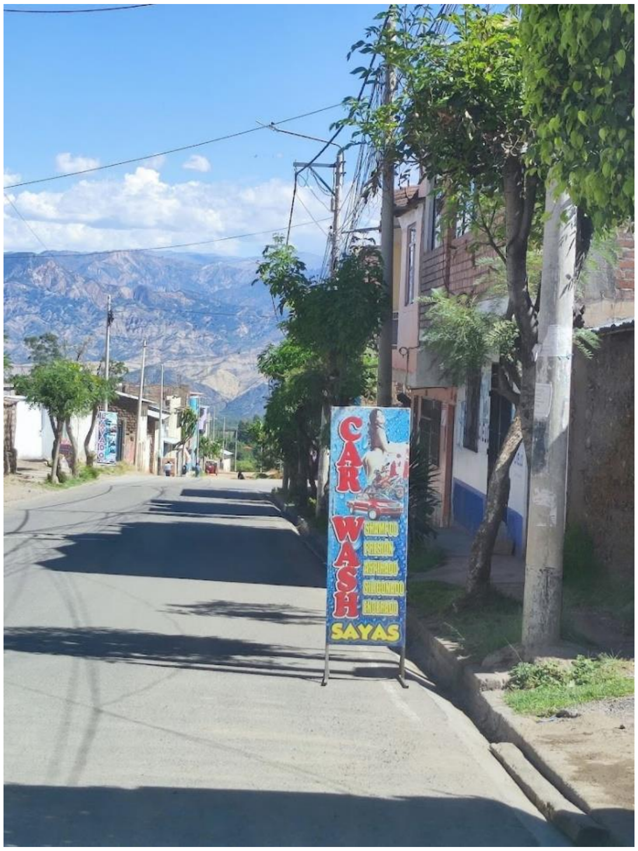
CAR WASH
SAYAS
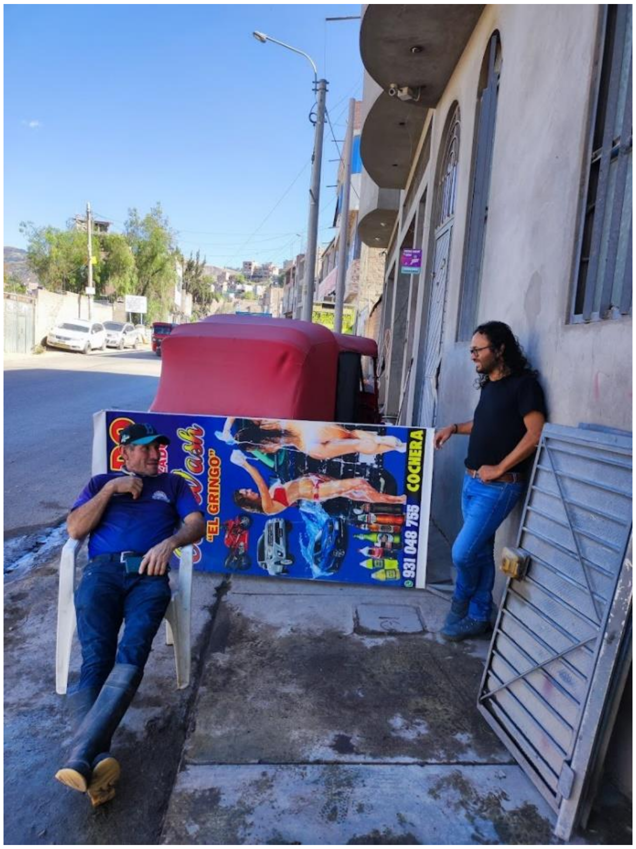
"EL GRINGO"
931 048 755
COCHERA

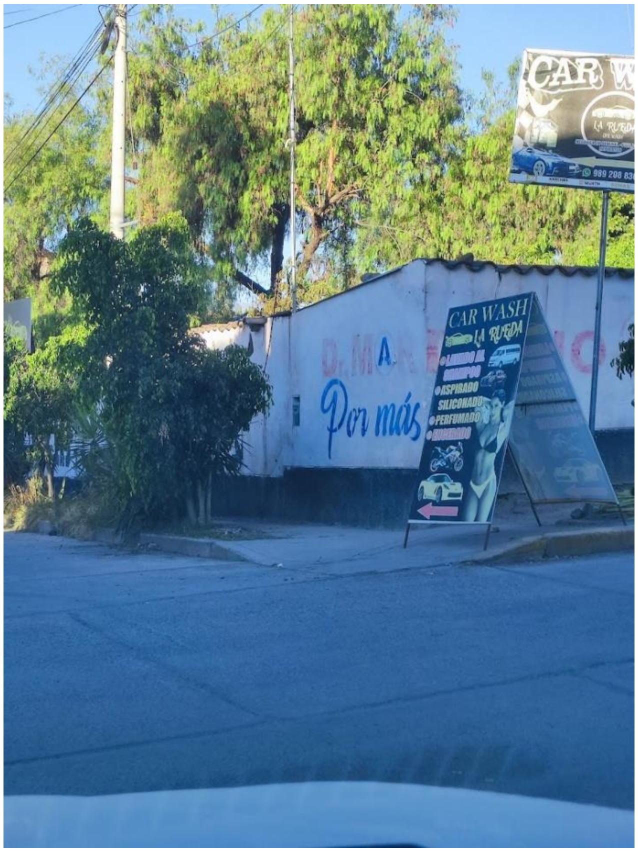
CAR WASH
LA RUEDA
ASPIRADO
SILICONADO
PERFUMADO
Por más
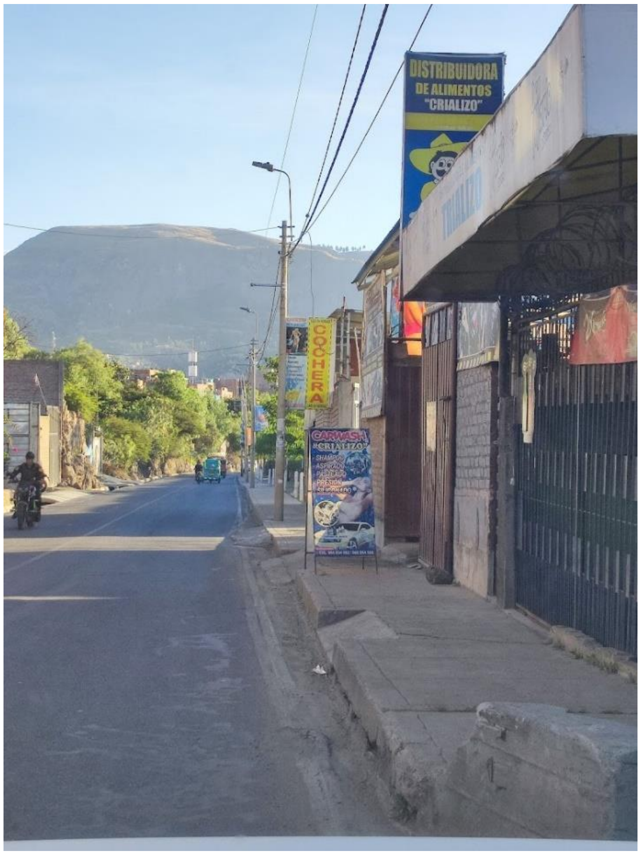
DISTRIBUIDORA
DE ALIMENTOS
"CRIALIZO"
COCHERA
CARWASH

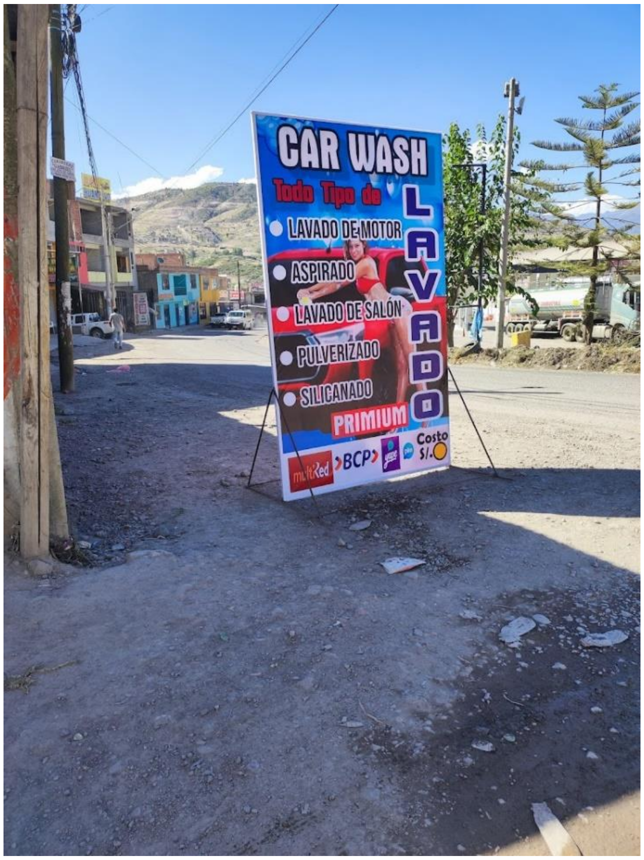
CAR WASH
Todo Tipo de
LAVADO DE MOTOR
ASPIRADO
LAVADO DE SALÓN
PULVERIZADO
SILICANADO
PRIMIUM
LAVADO
Costo S/.
BCP

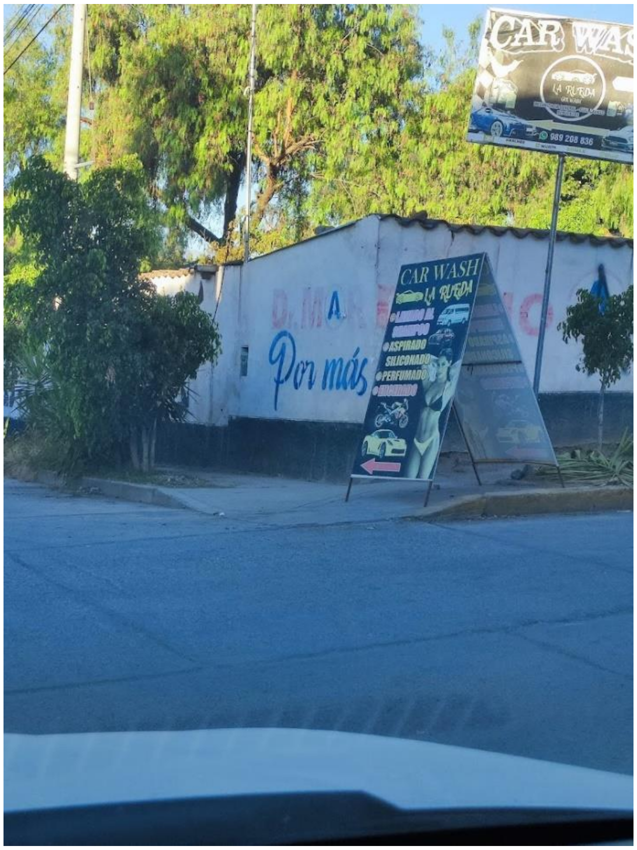
CAR WASH
LA RUEDA
CAR WASH
LA RUEDA
ASPIRADO
SILICONADO
PERFUMADO
Por más

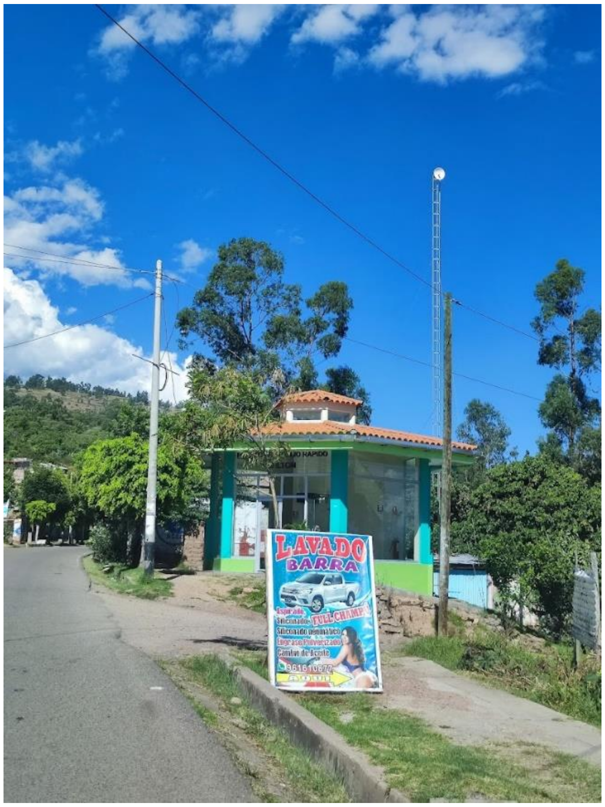
LAVADO
BARRA

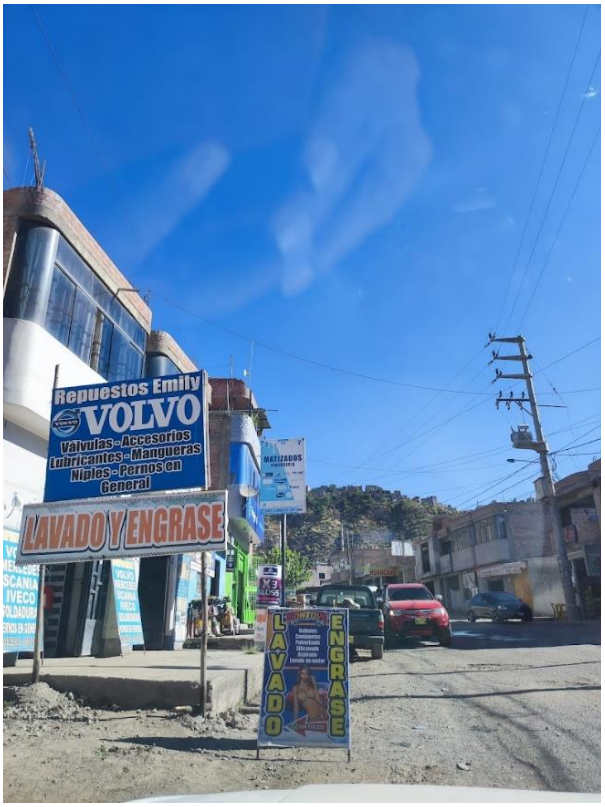
Repuestos Emily
VOLVO
Válvulas - Accesorios
Lubricantes - Mangueras
Niples - Pernos en
General
LAVADO Y ENGRASE
LAVADO
ENGRASE

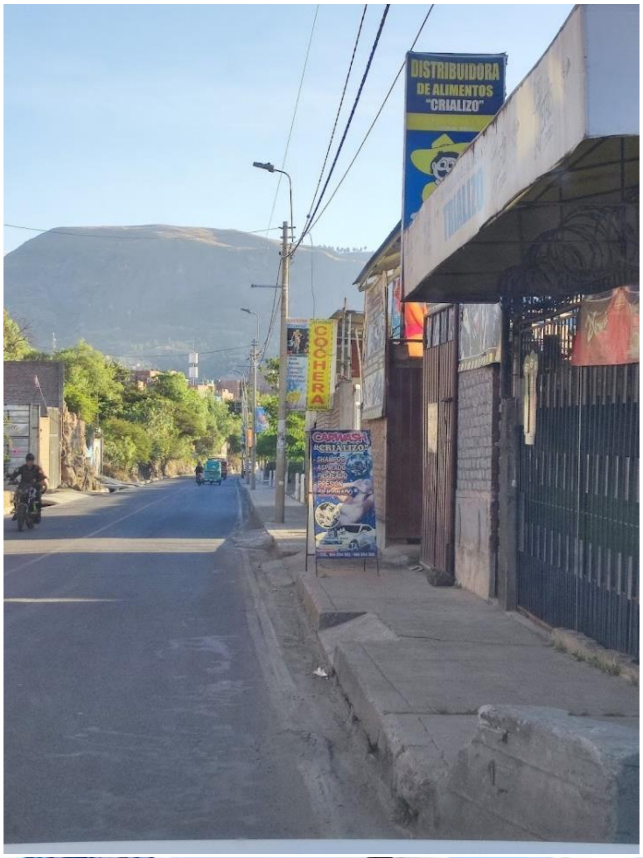
DISTRIBUIDORA
DE ALIMENTOS
"CRIALIZO"
COCHERA
CARWASH
"CRIALIZO"

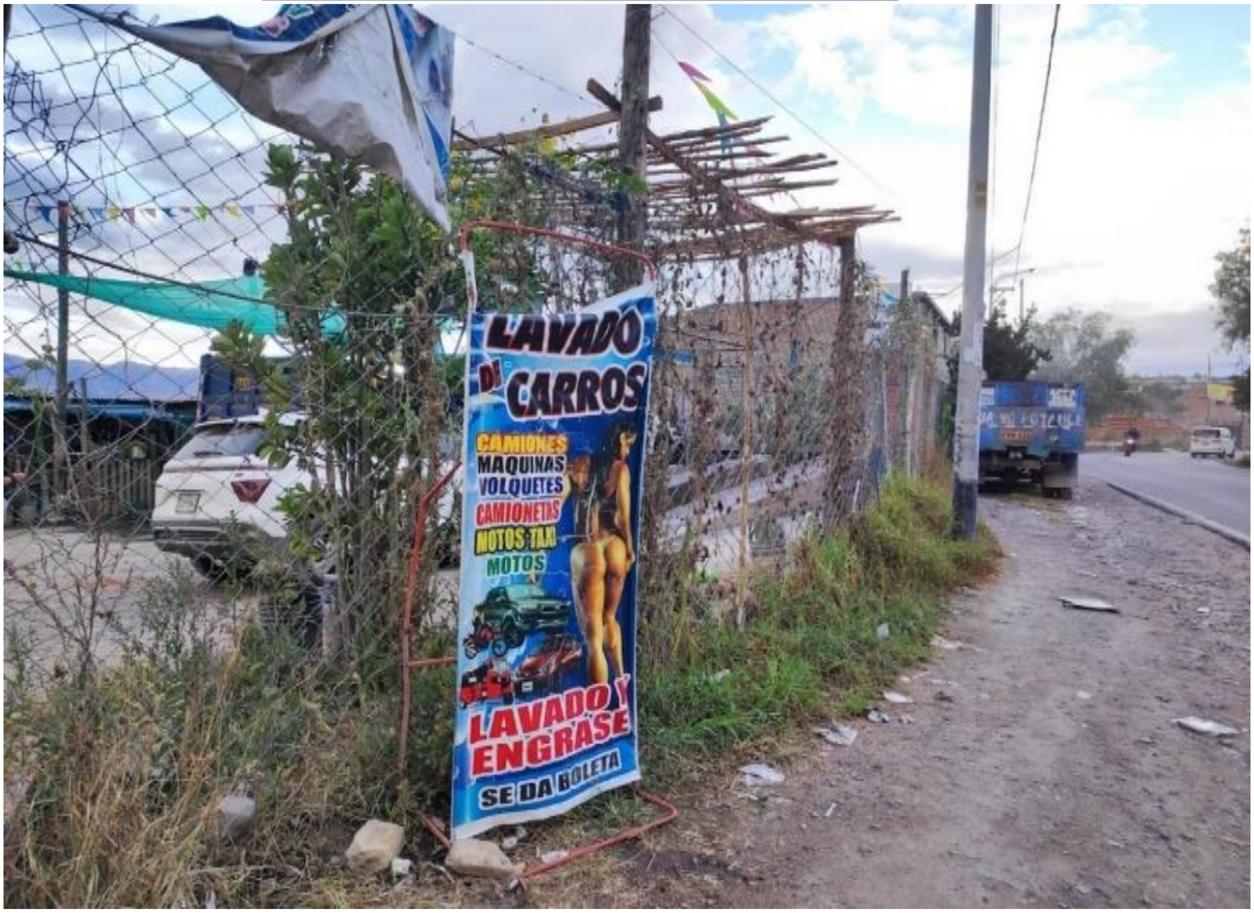
LAVADO
CARROS
CAMIONES
MAQUINAS
VOLQUETES
CAMIONETAS
MOTOS TAXI
MOTOS
LAVADO Y
ENGRASE
SE DA BOLETA

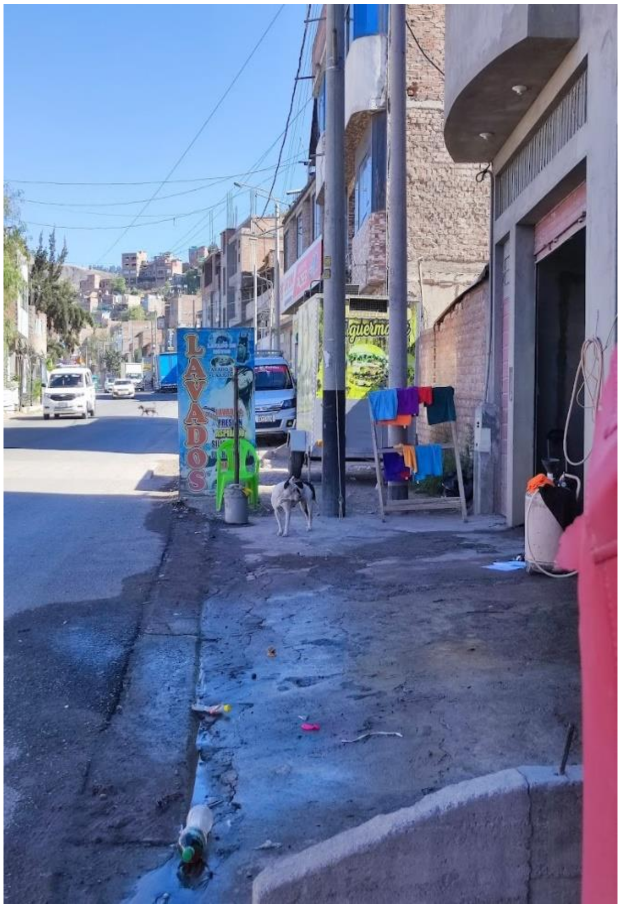
LAVADOS

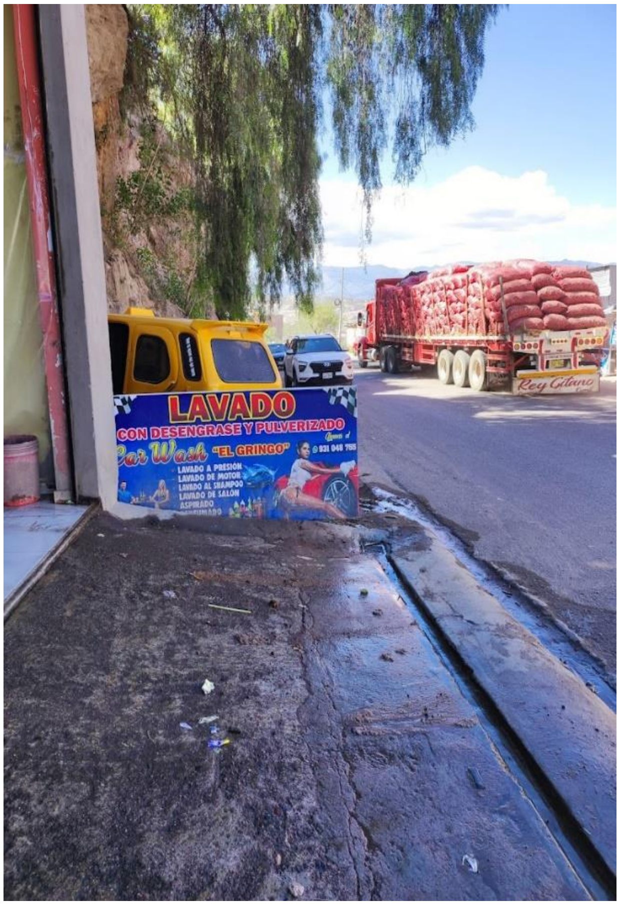
LAVADO
CON DESENGRASE Y PULVERIZADO
Car Wash "EL GRINGO"
Rey Gitano
